# Adipose-derived stem cells cooperate with fractional carbon dioxide laser in antagonizing photoaging: a potential role of Wnt and β-catenin signaling

**DOI:** 10.1186/2045-3701-4-24

**Published:** 2014-05-02

**Authors:** Xiao Xu, Hong-yi Wang, Yu Zhang, Yang Liu, Yan-qi Li, Kai Tao, Chu-Tse Wu, Ji-de Jin, Xiao-yan Liu

**Affiliations:** 1 Reconstructive and Plastic Surgery, The General Hospital of Shenyang Military Region, Shenyang, P.R. China; 2 Department of Experimental Hematology, Institute of Radiation Medicine, Beijing, P.R.China

**Keywords:** ADSCs, CO_2_ fractional laser, photoaging, Wnt/β-catenin signaling

## Abstract

**Background:**

It is well established that adipose-derived stem cells (ADSCs) produce and secrete cytokines/growth factors that antagonize UV-induced photoaging of skin. However, the exact molecular basis underlying the anti-photoaging effects exerted by ADSCs is not well understood, and whether ADSCs cooperate with fractional carbon dioxide (CO_2_) laser to facilitate photoaging skin healing process has not been explored. Here, we investigated the impacts of ADSCs on photoaging in a photoaging animal model, its associated mechanisms, and its functional cooperation with fractional CO_2_ laser in treatment of photoaging skin.

**Results:**

We showed that ADSCs improved dermal thickness and activated the proliferation of dermal fibroblast. We further demonstrated that the combined treatment of ADSCs and fractional CO_2_ laser, the latter which is often used to resurface skin and treat wrinkles, had more beneficial effects on the photoaging skin compared with each individual treatment. In our prepared HDF photoaging model, flow cytometry showed that, after adipose derived stem cells conditioned medium (ADSC-CM) co-cultured HDF photoaging model, the cell proliferation rate is higher than UVB irradiation induced HDF modeling (p < 0.05). Additionally, the expressions of β-catenin and Wnt3a, which were up-regulated after the transplantation of ADSCs alone or in combination with fractional CO_2_ laser treatment. And the expression of wnt3a and β-catenin has the positive correlation with photoaging related protein TGF-β2 and COLI. We also verified these protein expressions in tissue level. In addition, after injected SFRP2 into ADSC-CM co-cultured HDF photoaging model, wnt3a inhibitor, compared with un-intervened group, wnt3a, β-catenin protein level significantly decreased.

**Conclusion:**

Both ADSCs and fractional CO_2_ laser improved photoaging skin at least partially via targeting dermal fibroblast activity which was increased in photoaging skin. The combinatorial use of ADSCs and fractional CO_2_ laser synergistically improved the healing process of photoaging skin. Thus, we provide a strong rationale for a combined use of ADSCs and fractional CO_2_ laser in treatment of photoaging skin in clinic in the future. Moreover, we provided evidence that the Wnt/β-catenin signaling pathway may contribute to the activation of dermal fibroblast by the transplantation of ADSCs in both vitro and vivo experiment.

## Background

Solar ultraviolet (UV) irradiation causes premature aging of human skin, defined as photoaging, which is tightly associated with the increased occurrence of human skin cancer [1]. Given its importance in clinical and cosmetic fields, how to efficiently prevent photoaging and/or to treat photoaged skin has been a focus of research. One primary mechanism by which UV causes photoaging is the suppression of synthesis of type I procollagen (COLI) [2,3], a major structural protein in the skin connective tissue which is mainly produced by the fibroblasts located within dermis. Therefore, understanding how the generation of COLI is controlled, is one of the keys to develop effective therapeutic treatment which can be used to restore the expression of COLI in the photodamaged skin. The TGF-β/Smad pathway is the major regulator of synthesis of several components of the extracellular matrix, including type I and type III collagen by skin fibroblasts. It is TGF-β stimulates fibroblast proliferation in the dermis to enhance collagen synthesis [4-7]. Dermal thickness was reduced in TGF-β2 deficient mice, but not in TGF-β1 and TGF-β3 deficient mice [8]. Previous studies suggested that a number of signaling pathways govern the production of COLI [9-11], one of which is the highly conserved Wnt/β-catenin pathway. Wnt molecules affect cell membrane through paracrine and autocrine functions. So far, among known Wnt protein family members, Wnt1 [12,13], Wnt3a [14] and Wnt8 [15] are able to activate classical Wnt-β-catenin-LEF/TCF pathway, and Wnt3a upregulates TGF-β in a β-catenin dependent manner through Smad2, and inducts the differentiation of myofibroblast [16].

Wnt/β-catenin signaling integrates signals from numerous signaling pathways including TGF-β and FGF to mediate a variety of cellular activities including cell proliferation and differentiation, suggesting that Wnt/β-catenin plays important roles in mediating COLI production and skin damage through TGF-β.

Adipose-derived stem cells (ADSCs) exhibit the ability to self-renew, proliferate and differentiate into multiple lineage-specific cells in response to different stimuli, therefore, is an ideal source for tissue engineering and regenerative medicine. Indeed, ADSCs and its conditioned medium (CM) that contains a variety of cytokines and growth factors [17], facilitated the wound healing processes by stimulating collagen synthesis in both vitro and vivo, such as in micropig and human patient [18,19]. As one of five major growth factor families have been studied with regard to the wound-healing process, ADSCs secreted growth factor, TGF-β, it appears to be the most potent stimulator to collagen remodeling by fibroblasts [20,21]. However, the molecular basis underpinning the reduced UV exposure-induced skin damage by ADSCs application is not well understood.

Another effective way for the treatment of pathological conditions of skin such as photoaging skin is the application of fractional CO_2_ laser, which has been well documented [22-24]. A combined use of fractional CO_2_ laser and other skin damage treatments such as radiofrequency waves was also proposed in order to achieve the best efficacy of treatment [25], however, whether the application of ADSCs and the use of fractional CO_2_ laser can be combined in clinical use had not been realized until recently. Zhou BR et al. reported that sequential use of fractional CO_2_ laser followed by ADSCs-CM enhanced wound healing [26]. However, the molecular mechanisms underlying the improved benefits achieved by the combined use of these different treatments remain enigmatic.

In the present study, we investigated the impacts of ADSCs alone or in combination with fractional CO_2_ laser on photoaging skin caused by UV irradiation in the animal model, and report here that ADSCs improved photoaging skin recovery at least partially via restoring the expression of β-catenin and Wnt3a, and the combined use of ADSCs transplantation and fractional CO_2_ laser synergistically benefited the UV-irradiation induced skin damage compared with either individual treatment. At the same time, we observed that compared with UVB irradiation induced HDF photoaging model, ADSC-CM co-cultured photoaging HDF improved the cell cycle arrest, and verified protein expression of wnt3a and β-catenin in injected SFRP2 to ADSC-CM co-cultured photoaging HDF before and after, have positive correlation with the photoaging associated protein TGF-β2 and COLI. Thus, we presented a pilot study to provide a strong rationale for a combined use of ADSCs and fractional CO_2_ laser in the clinic in the future.

## Results

### ADSCs or fractional CO_2_ laser treatment improved histological formation of the photoaging skin induced by UVB irradiation

**Figure 1:**
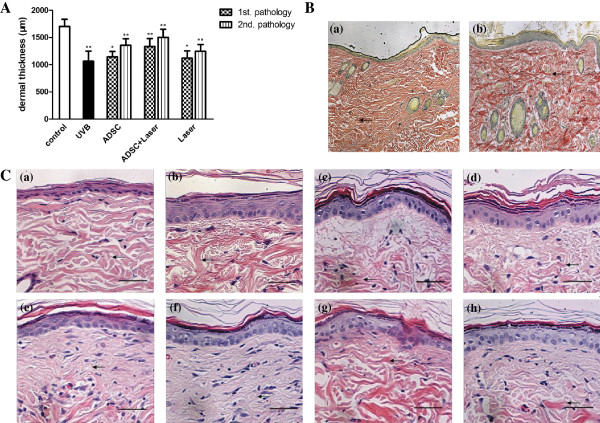
**Skin histopathology evaluation. A.** ADSCs or fractional CO_2_ laser treatment improved dermal thickness in the photoaging skin induced by UVB irradiation. **B.** V&G staining shows the light red collagen fibers(* indicate) decreased,dyeing shallowed,especially in the superficial layer dermis,however,the dark red elastic fiber(arrows indicate) increased, thicker, partial hyperplastic elastic fibers were fractured and snarling after UVB irradiation. ×400. Scale bars are 100 μm. **C.** fibers were stianed red,and that improved the enlarged diameter metamorphic fizzy fibers with infiltration of inflammatory cells in ADSC transplantation group **(c,d)** and CO_2_ fractional laser treatment group **(g,h)**. The improvement effect was significantly increased in combined treatment group **(e,f)**. H&E × 400. Scale bars are 100 μm.

H&E staining showed that the normal group (Figure 1Ca) epidermal layer formation was unbroken, that the cell level was clear, and that the thickness was normal. The dermis layer was full of wavy fiber tissues which was well-proportioned. The UVB induced epidermal layer incrassation and hyperkeratosis in irradiation group (Figure 1Cb), and the cell level was unclearness and the dermis layer fiber was not well-proportioned. The transverse section showed enlarged diameter of the collagen fiber with infiltration of inflammatory cells. We also found that the dermal thickness of the three treatment group increased obviously (Figure 1A). COLI was increased also, mainly in the newborn slender collagen fiber and in the metamorphic frizzy fiber. UVB-irradiated rat showed great changes in skin appendages and the effect of treatment by ADSCs and CO_2_ fractional laser on dermal thickness and in UVB-irradiated rat were investigated. Measurement of the dermal thickness (Figure 1A) showed great decrease after UVB irradiation in rat photoaging model; however, it showed significant increase in the groups of ADSCs transplantation, carbon dioxide fractional laser, especially after the combined treatment of ADSCs and CO_2_ fractional laser at the second pathology. VG staining (Figure 1B) showed that, compared with the normal group, the collagen fiber content of irradiation group reduced, dyeing lighter, especially in the dermal superficial layer. The elastic fibers increased and became thicker, partial hyperplastic elastic fibers fractured and snarled. HE stained and VG stained (Figure 1C and B) sections obtained from the animals treated with the vehicle alone showed a significant increase in epidermal thickness because of UVB irradiation for 8 weeks. This finding was consistent with the previously reported finding that significant epidermal hyperplasia was induced during photoaging due to UVB irradiation [27]. However, compared with the rat treated with ADSCs (Figure 1c, d) and CO_2_ fractional laser (Figure 1Cg, h), the epidermal hyperplasia of rat, which was treated with combined treatment of ADSCs and Fractional laser, was obviously diminished (Figure 1Ce, f). Above all, it illustrated that ADSCs transplantation and CO_2_ fractional laser treatment could improved UVB-irradiated induced photoaging, and could get better combined utilization.

### COLI immunohistology staining in pretreatment and post-treatment

**Figure 2:**
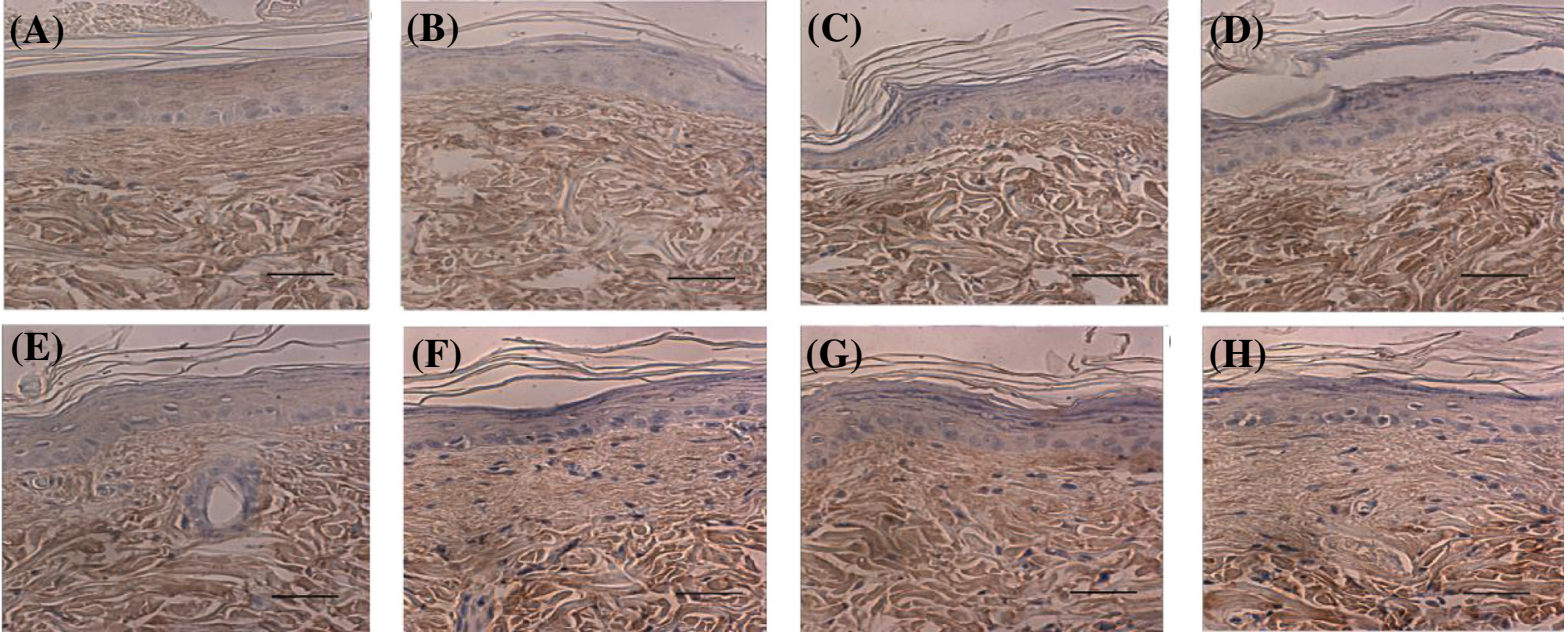
**UV irradiation alters COLI protein expression in rat skin in vivo - immunohistology.** Biopsied skin samples were obtained 8 weeks after exposure to UVB, and were localized by immunohistology, as described in Materials and Methods. **A**, non-irradiated skin; **B**, UVB-irradiated induced photoaging skin; **C**, ADSCs pretreatment after 7 d; **D**, ADSCs pretreatment after 14 d; **E**, combined treatment group after 7 d; **F**, combined treatment group after 14 d; **G**, CO_2_ fractional laser pretreatment after 7 d; **H**, CO_2_ fractional laser pretreatment after 14 d. Panels show results for a single individual, but are representative of six individuals. Immunostain, ×400. Scale bars are 100 μm.

COLI immunohistology staining weaken after UVB irradiation induced photoaging skin, and enhanced obviously after three treatment group, especially for the combined group. As reported, the vivo –immunohistology staining (Figure 2) showed that the increased COLI mainly appeared in the dermal full-thickness, especially for the superficial layer, the place where COLI increased is in keeping with the place where COLI reduced in photoaging [28]. After the treatment, the most obvious place that collagen increased is appeared in the dermal superficial layer Grenz area [29].

### ADSCs repressed MDA production, and restored the activities of total SOD

**Figure 3:**
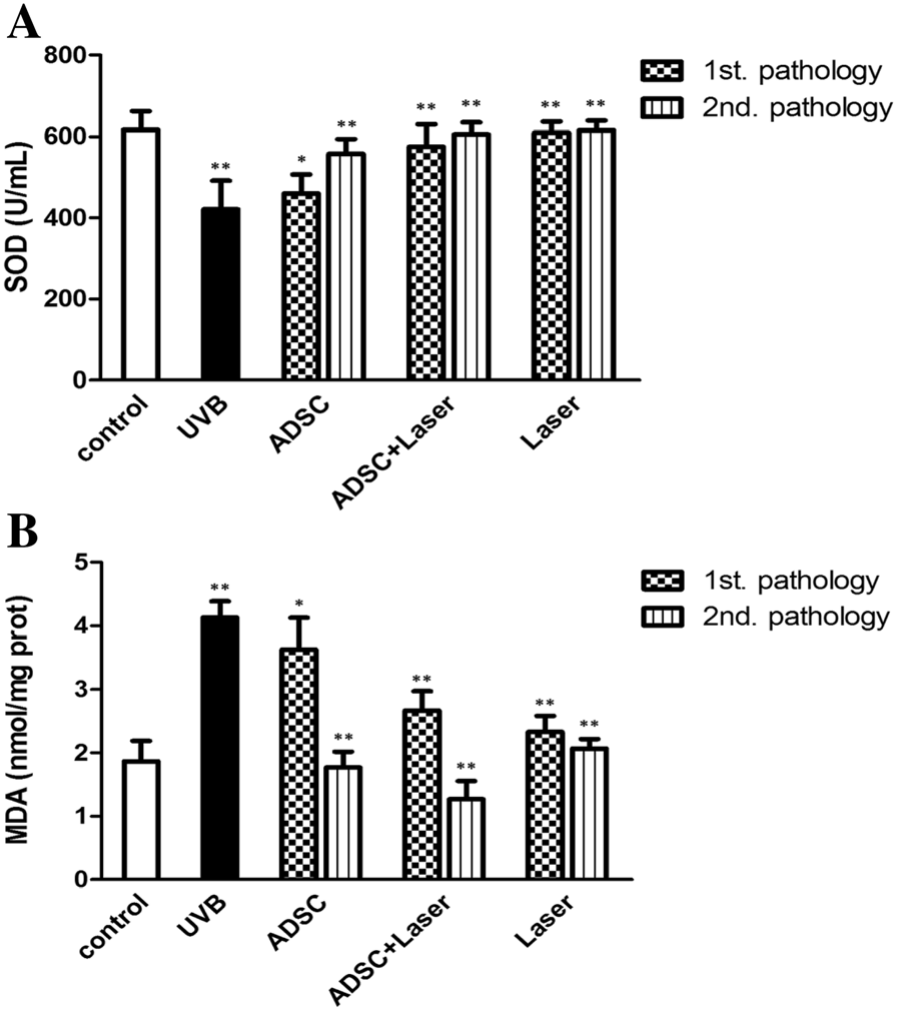
**ADSCs repressed MDA production, and restored the activity of total SOD. A.** Treatment of ADSCs or fractional CO_2_ laser, or in combination, decreased MDA content in the skin irradiated by UVB. **B.** Treatment of ADSCs or fractional CO_2_ laser, or in combination, elevated SOD levels in the skin irradiated by UVB.

One of the primary molecules that are implicated in photoaging is the oxygen free radicals, which are produced through enzyme and non-enzyme system. The overproduction of the oxygen free radicals under pathological conditions leads to the formation of lipid peroxides such as MDA, consequently causing tissue photoaging. As shown in Figure 3, UVB irradiation increased MDA content in the control groups (p < 0.01). In contrast, either ADSCs or fractional CO_2_ treatment significantly reduced MDA content, and the combined use of these two further decreased it. Elimination of free radicals is dependent on the preventive or interrupted regulations of antioxidant defense system. SOD is an important component of the antioxidant system, which removes superoxides and protects cells from damage. As shown in Figure 3, UVB irradiation significantly decreased SOD, while ADSCs or fractional CO_2_ laser treatment alone substantially restored the level of SOD equivalent to that of the control group. Intriguingly, the ADSCs and fractional CO_2_ laser did not show cooperative effects on SOD level compared with each single treatment. Collectively, these data suggest that ADSCs or fractional CO_2_ laser improved wound healing at least partially targets free radicals, and that combinatorial use of these two cooperatively inhibits MDA production, but not synergistically increases SOD levels.

### Survival of ADSCs

**Figure 4:**
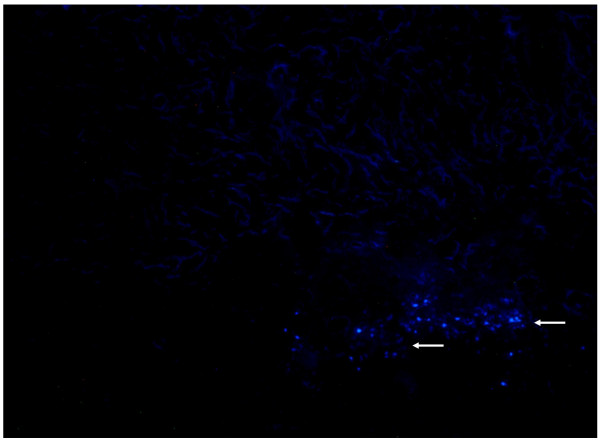
**Survival of ADSC.** ADSCs were stained with DAPI and were directly injected in the skin of rat. Two weeks after injection, rat skin block was made using cryosection and counterstained with blue-fluorescent nucleic acid stain. ADSCs are stained blue (arrows indicate).

As cell transplantation between species mediates immune reject responses, the survival of ADSCs from humans was investigated after transplantation of ADSCs labeled with DAPI. After two weeks of transplantation, a rat skin block was made using cryosection. ADSCs with DAPI staining could be seen at the deep layer of dermal after 1 week. However, the fluorescence became weaken after two weeks, as shown in Figure 4. Although the survival rate after ADSCs transplantation was not measured, human ADSC may survive and improve skin photoaging in rat at least for two weeks.

### Sustained upregulation of TGF-β2, wnt3a and β-catenin in dermal tissue leads to an increase and the activation of dermal fibroblasts by westernblot

**Figure 5:**
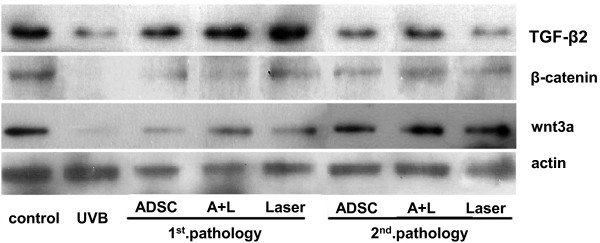
**Differential expression of wnt/β-catenin signaling pathway protein and TGF- β2 during photoaging treatment.** Expression of wnt3a and β-catenin were upregulated by ADSC transplantation and CO_2_ fractional laser treatment.Expression of TGF-β2 was upregulated during photoaging treatment.

Since protein expressions of TGF-β2, wnt3a and β-catenin were upregulated in UVB irradiated rat tissue after the treatment of ADSCs and CO_2_ fractional laser (Figure 5), collagen contents in the dermis were significantly increased in treated rat (Figure 2), especially for the combined treatment group. We observed that the change of Wnt3a and β-catenin expression followed the change of TGF-β2 expression. As we hypothesized, β-catenin and wnt3a expressions were upregulated after the ADSCs transplantation, and were more significantly up regulated after the second pathology. Interestingly they also had increased expression in the other two groups, combined treatment of ADSCs with the carbon dioxide fractional laser treatment, and carbon dioxide fractional laser only. These results indicate that increased collagen contents in the dermis of treated rats are at least partially mediated by the restored expression of β-catenin and wnt3a.

### ADSC reduced cell cycle arrest of HDF induced by UVB irradiation

**Figure 6:**
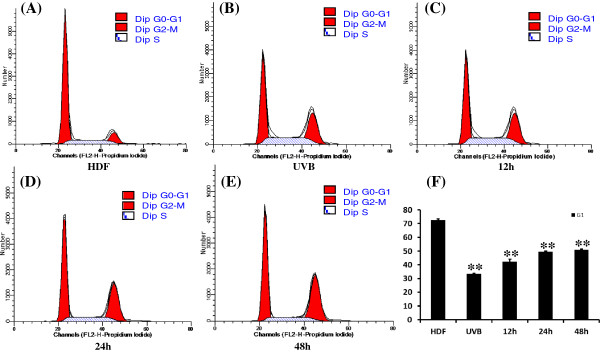
**Cell cycle analysis of DNA contents.** Untreated HDF showed more G1 phases **(A)**. However, UVB irradiation significantly decreased G1 (proliferation) cells **(B)**, which were reversed by ADSC-CM pretreatment from 12h **(C)**, 24h **(D)** to 48h **(E)**. And compared with photoaging model HDF, the ADSC-CM significantly increased the cell proliferation rate until 48h **(F)**.

As UVB irradiation induced cell cycle arrest, cell cycle analysis was performed after ADSC-CM pretreatment. UVB irradiation decreased the G1 phase of the HDF cells, which was reversed by ADSC-CM pretreatment (Figure 6). These results indicate that ADSC-CM decreased the cell cycle arrest in HDF induced by UVB irradiation.

### Protein expression of Wnt3a and β-catenin and photoaging related protein

**Figure 7:**
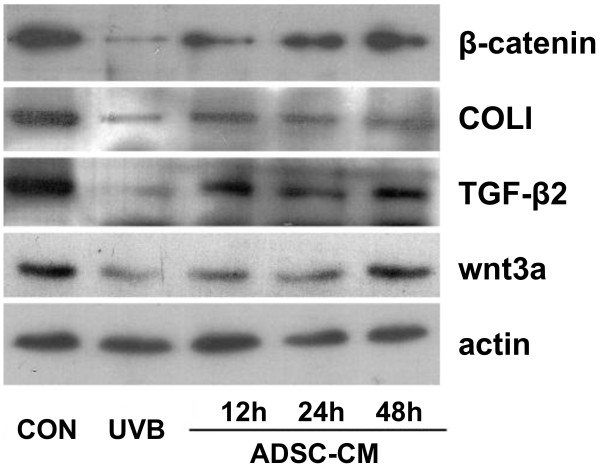
**Effect of ADSC-CM on the expression of wnt/β-catenin signaling pathway proteins and photoaging relevant proteins.** ADSC-CM significantly increased the protein expression of wnt3a and β-catenin from 24 h, expression of TGF-β2 and COLI were upregulated as well.

To assess ADSC-CM induced modulation of protein expression level, westernblot was performed on wnt3a and β-catenin and photoaging related protein TGF-β2 and COLI (Figure 7). UVB irradiation clearly reduced the expression of wnt3a and β-catenin, and reduced that of TGF-β2 and COLI.However, the expression of wnt3a and β-catenin were significantly increased after ADSC-CM pretreatment after 24 h, while that of TGF-β2 and COLI were upregulated after ADSC-CM pretreatment. It is reasonable to conclude that ADSC-CM does accelerate the expression of wnt3a and β-catenin in treated photoaging HDFs, and the expression of TGF-β2 has positive correlation with wnt3a and β-catenin.

### Effect of different density SFRP2 to the expression of wnt3a and β-catenin in processing of ADSC-CM treated photoaging HDF

**Figure 8:**
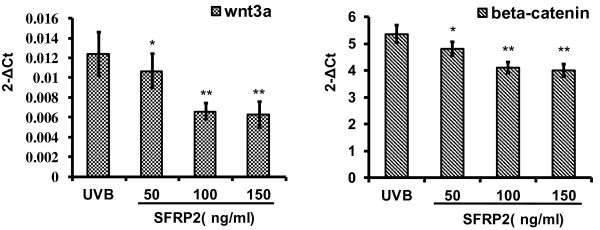
**Effect of different density SFRP2 on the expression of wnt/β-catenin signaling proteins in ADSC-CM cultured photoaging HDF.** 100 ng/ml SFRP2 significantly down regulated the expression of wnt3a and β-catenin.

In vitro, as wnt3a inhibitor, SFRP2 injected into ADSC-CM co-cultured photoaging HDF, set the final concentration respectively, 50 ng/ml, 100 ng/ml, 150 ng/ml. The result showed that, 100 ng/ml SFRP2 has maximum intervene effect on wnt3a and β-catenin of ADSC-CM co-cultured photoaging HDF in mRNA level. And then following the increased SFRP2 concentration, wnt3a and β-catenin still keep the constant level (Figure 8).

### Effect of Reduced wnt3a to the protein expression of wnt/β-catenin signaling pathway in ADSC-CM co-cultured photoaging HDFs

**Figure 9:**
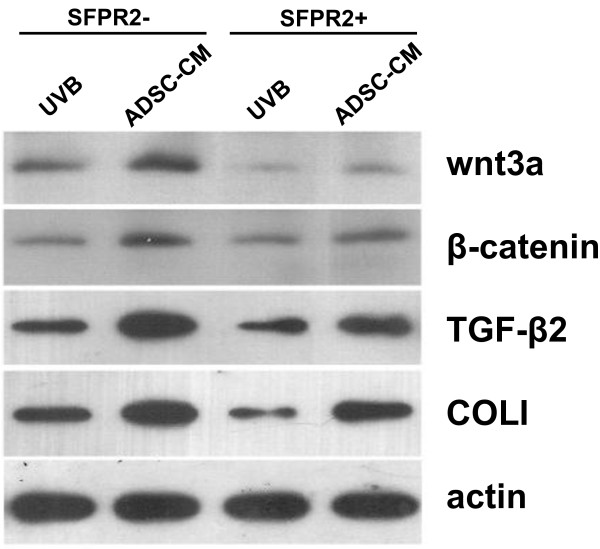
**Effect of intervened expression of wnt/β-catenin signaling pathway proteins during ADSC-CM treated photoaging HDF.** Down regulated expression of wnt3a and β-catenin inhibited the effect of ADSC-CM treated photoaging HDF.

In the experiments, wnt3a inhibitor SFRP2 was applied to H-DMEM, which is used to culture photoaging HDF, compared with unaffected group, the protein expression levels of wnt3a, β-catenin, TGF-β2 and COLI were all down regulated in different degrees, wnt3a and COLI were sharply downregulated in particular (**, p < 0.01) (Figure 9). SFRP2 was applied to ADSC-CM, which is used to culture photoaging HDF, compared with unaffected group, the protein expression levels of β-catenin, TGF-β2 and COLI were down regulated, and the differences have statistical significance, wnt3a level decreased in particular (**, p < 0.01).

## Discussion

In the present report, we not only provided data showing that ADSCs or fractional CO_2_ laser improved the photodamaged skin induced by UVB exposure in the animal model, but also further demonstrated that the synergistic beneficial actions rendered by the combinatorial use of these two treatments, as evidenced by more dermal thickening compared with that obtained by either single treatment.

UVB-induced photoaging skin was better improved by the combined use of ADSCs transplantation and CO_2_ fractional laser treatment, compared with either of these treatments. ADSCs and secreted soluble factors showed promise for the treatment of photoaging [30]. Photoaging is a kind of wound, our modeling made a dual wound caused by UVB induced irradiation and CO_2_ fractional laser, an artificial wound. And then injected with the ADSCs before the wound heals, conditioned medium from ADSCs (ADSC-CM) significantly stimulated both collagen synthesis and migration of dermal fibroblasts, give play to more treatment effect.

Previous studies suggested that photoaging had pathological processes that were similar to that of skin wounds, in which dermal fibroblasts play critical roles via interaction with other type of cells including fat and mast cells [31]. Dermal fibroblasts also produced numerous proteins that are important to main normal skin function [32], among which is COLI. Studies confirmed that CM from ADSCs, which secreted a number of cytokines including platelet-derived growth factor, vascular endothelial growth factor [30,33], promoted synthesis of COLI in cultured fibroblasts. Thus, the benefit of ADSCs to improve wound healing processes is at least partially linked to the elevated production of COLI.

It is widely believed that one of the major contributors to the UV-induced skin damage is reactive oxygen species (ROS) [34], which repressed anti-oxidative defense system, consequently resulting in premature skin aging and other pathological phenotypes [35]. A variety of anti-oxidative agents such as vitamin C and E were proved useful in anti-UV-induced skin damage [36,37]. Indeed, our studies suggested that MDA, one of the major products generated by oxygen free radicals, were dramatically increased in the UVB-damaged skin. However, both ADSCs and fractional CO_2_ laser treatment reversed this increase. More interestingly, the combined use of these two applications, which was rarely explored before, synergistically suppressed the increase of MDA content in the photoaging skin. On the other hand, the levels of SOD, a well-known scavenger of free radicals, were significantly recovered by either of these two treatments to the levels that were equivalent to those detected in the normal skin tissue. However, no cooperative effect of ADSCs and fractional CO_2_ laser on the levels of SOD in the damaged skin was observed compared with each single treatment, suggestive of that the observed synergy between ADSCs and fractional CO_2_ laser in antioxidative stress, i.e. decreasing MDA content, in the diseased skin imposed by UV irradiation, was achieved via SOD-independent mechanism.

Multiple signal transduction pathways converge on Wnt/β-catenin signaling to mediate cell proliferation, differentiation and development in a variety of tissues or organs, which plays important roles in generation of tissues, including skin [38,39]. Potentiating endogenous Wnt/β-catenin signaling promoted skin wound healing [40], which was partially attributable to the elevated proliferation, migration and local invasion of fibroblasts [41]. Thus, the activity of wnt3a and β-catenin in Wnt/ β -catenin signaling may be important for TGF-β2 to production of COLI. Indeed, our findings indicated that UV-induced skin damage was accompanied with decreased expressions of Wnt/β-catenin, which was partially restored by ADSCs. Thus, we believe that one of mechanisms by which ADSCs treatment improved photoaging skin was the rescued activity of Wnt/β-catenin signaling.

## Conclusion

We presented evidence that ADSCs transplantation or fractional CO_2_ laser treatment improved UV-induced skin photoaging, and that combined use of both treatments synergistically improved photoaging skin. Mechanistically, ADSCs transplantation promoted recovery of the photodamaged skin at least via upregulate perturbed Wnt/β-catenin signaling imposed by UV irradiation to activate TGF-β2. However, given the complexity of photoaging processes that may implicate a multitude of signaling pathways and a variety of molecules, the involvement of other molecular mechanisms that also contribute to the more beneficial effects observed with the use of ADSCs transplantation deserves or needs further investigation.

## Materials and methods

### Isolation and culture of ADSC

Human subcutaneous adipose tissue samples were acquired from elective liposuction of healthy females with informed consents as approved by the institutional review boards. The adipose tissue samples were centrifuged at 100 g for 3 min, and the obtained samples were digested with 1% collagenase type II (Sigma–Aldrich, St. Louis, MO) and 0.15% Trypsin under gentle agitation for 40 min at 200 rpm at 37°C, and centrifuged at 700 g for 5 min to obtain the stromal cell fraction. The pellet was filtered with 70 mm nylon mesh filter, and resuspended in 1× phosphate buffered saline (PBS). The cell suspension was layered onto histopaque-1077 (Sigma–Aldrich, St. Louis, MO), and centrifuged at 700 g for 5 min. The supernatant was discarded, and the cell band buoyant over histopaque was collected. The retrieved cell fraction was cultured overnight at 37°C/5% CO_2_ in the control medium Dulbecco’s modified Eagle media (DMEM), supplemented with 10% fetal bovine serum (FBS), 100 units/ml of penicillin, and 100 mg/ml of streptomycin. The resulting cell population was maintained over three to five days until confluence. ADSCs were cultured and expanded in the control medium. The General Hospital of Shenyang Military Region ethical committee.

### Generation of photoaging animal model

20 male and 20 female, six to eight-week-old SD rats were provided by Beijing Institute of Radiation Medicine. All rats were housed in the climate-controlled quarters (22 ± 1°C with 50% humidity) with a 12/12 h light/dark cycle. Animals were allowed free access to water and chow diets and were observed daily. The rats were irradiated dorsally using the UVB-emitting system 40 W (LEITUO illumination, Shenzhen, China) for eight weeks. The peak of emission near 312 nm, the irradiance between 290 and 320 nm corresponding to 55% of the total amount of UVB. The distance from the lamps to the animals’ backs was about 35–40 cm. During exposure, the animals could move around freely in their cages. The irradiation dose was one MED (minimal erythemal dose; 30 mJ/cm^2^) in the first two weeks, two MED (40 mJ/cm^2^) in the third week, three MED in the fourth week (50 mJ/cm^2^), and four MED (60 mJ/cm^2^) in the fifth through eight weeks. The total UVB dose was approximately 115 MED (7.4 J/cm^2^).

### Animal grouping

After photoaging induction, the animals were divided into the following groups: 1) UVB group: 1 ml of PBS was injected; 2) the 1^st^ treatment group: ADSCs (1 × 10^7^), which were suspended in 1 ml 1 × PBS, were subcutaneously injected into the restricted area of the rats two times in every seven-day interval; 3) the 2^nd^ treatment group: in combination with CO_2_ laser (King, JiLin, China. Energy: 10 J/CM^2^; density: 9.6; degree: 3; spot size: 1.3 mm, pattern: square). CO_2_ laser was used to treat the damaged skin area two times every turn as the skin began to appear the white spot. After then, ADSCs were injected to the area which had been treated by the fractional CO_2_ laser; 4) The 3^rd^ treatment group: only fractional CO_2_ laser treatment. The control group is the one that had no photodamaging skin. Skin samples from all these treatment groups were cut every seven days after the second treatment and were weighted. Some cut skins were fixed in formalin solution for histologial examination.

### Survival of ADSCs

Blue fluorescent-labeled ADSCs were transplanted to examine the survival of ADSCs. Suspended ADSCs (1× 10^5^ cells/cm^2^) were labeled with 50 μg/ml fluorescent dye (DAPI, Sigma, Saint Louis, MO.). One hour after labeling, FBS was added for 1 min to stop the reaction and the cells were washed by PBS. The sensitivity and specificity for cell labeling with DIPA was almost 100%. Then, DAPI-labeled ADSCs were subcutaneously injected into the notum skin of rat photoaging model (1 cm^2^ × 1 cm^2^). Every 7 days after experiment, frozen sections of the skin appendages were prepared.

### Antioxidant capacity

Superoxide dismutase (SOD) activities were determined using commercially available kits. Total SOD (T-SOD) activity was determined through xanthine oxidase method [42], and the data was expressed by U/mL nitrite unit. MDA content was measured using thiobarbituric acid (TBA) method at absorbance of 532 nm [43], and the data was expressed by nmol/mL protein. All procedures were performed with assay kits according to the manufacturer’s instructions.

### Tissue histology

Dorsal skins (1.5 cm × 1.5 cm) were fixed in 10% formalin neutral buffered solution, embedded in polyester wax and sectioned at 6 mm. The sections were subjected to Hematoxylin & Eosin (H&E) and Van Gieson (V&G) staining.

### HDF culture and UVB induced irradiation

HDFs were cultured in a HIGH-DMEM supplemented with 10% fetal bovine serum, 100U/ml penicillin and 100 μg/ml streptomycin in 5% CO_2_ at 37°C. After starvation with serum-free medium for 24 h, cells were washed with PBS and exposed to UVB with 3–4 drops PBS. UVB irradiation was carried out using a UV lighter (LEITUO illumination, Shenzhen, China). Immediately after the irradiation, the PBS was aspirated and replaced with complete medium. UVB irradiation doses were tested in 30–60 mJ/cm^2^ and finally fixed to be 50 mJ/cm^2^ for further experiment. The irradiation lasted 50 min per day. And it was totally 5 days.

### Preparation of ADSC-CM

ADSCs (4 × 10^5^ cells) were cultured in H-DMEM serum-free medium. Conditioned medium of ADSCs was collected after 72 h of culture, centrifuged at 1500 rpm for 5 min and filtered using a 0.22 μm syringe filter. ADSC-CM co-cultured UVB irradiation induced HDF photoaging model, after 12 h, 24 h and 48 h, digested the co-cultured cell photoaging model, standby application.

### Cell cycle analysis by flow cytometry

HDFs (2 × 10^5^) were seeded in 100 mm dishes, incubated and allows growing to 60% confluency. After starvation with serum-free medium for 24 h, the cells were exposed to UVB (50 mJ/cm^2^) for 50 min every day, until the fifth day, continuously cultured for 12 h, 24 h and 48 h with ADSC-CM. And then, UVB-irradiated HDFs were cultured in complete medium for 24 h, harvested, washed twice with PBS, and permeabilized with 70% ethanol at 0°C before analysis. The cells were then washed twice with PBS-treated RNAse (30 min at 37°C, 1 mg/ml). Cellular DNA was stained with 100 mg/ml propidium iodide. The distribution of cell cycle phases with different DNA contents was read in a FACScan flow cytometer (Becton–Dickinson, San Jose, CA).

### Quantitative Real-Time RT-PCR

Total RNA was isolated using TRIzol reagent (Sigma, USA) according to the manufacturer's instructions. The RNA was quantified with a NanoDrop ND-1000 Spectrophotometer (NanoDrop Technologies, Wilmington, USA) and analyzed by RNA agarose gel electrophoresis. The RNA was reverse transcribed by using the high capacity cDNA reverse transcription kit (Applied Biosystems) and the experiment was performed with an RNA PCR kit (TAKARA, Japan). Quantification of mRNA expression was performed by real-time quantitative PCR (Q-PCR) using the ABI PRISM 7500FAST Sequence Detection System Instrument (Applied Biosystems, Applera Dcutschl and GmbH, Darmstadt, Germany).

The PCR reactions for wnt3a, β-catenin and β-actin mRNA (94°C for 30 s, 54°C for 45 s, 72°C for 1 min, 35 cycles) were carried out using the following forward and reverse primers: Wnt3a, forward 5’ - GCC CCA CTC GGA TAC TTC TT- 3’, reverse 5’- CAC TCC TGG ATG CCA ATC TT- 3’; β-catenin, forward 5’- AAC GGC TTT CGG TTG AGC TG - 3’, reverse 5’- AGG TTG CTA CCG CTG AGT CC- 3’; β-actin, Forward 5' - CCT GGC ACC CAG CAC AAT- 3', Reverse 5' - GGG CCG GAC TCG TCA TAC- 3'. Standard curves showed that PCR efficiency was 98 - 100% for the assays. Negative controls, such as cDNA reactions without RT or RNA, and PCR mixtures lacking cDNA were included to detect possible contamination. Melt curve analysis was conducted to confirm reaction specificity. Samples were quantified by the relative standard curve method using standard curves made from serial dilutions of interest gene plasmid standards.

### Western blot analysis

In short, the rat dermal samples or UVB irradiation induced HDF selected the same methods, lysed in RIPA buffer and protein lysates were separated on SDS polyacryamide gel by electrophoresis. The proteins were transferred to PVDF membranes, and then blocked by 5% nonfat milk at 4°C overnight. Membranes were incubated with antibodies of Wnt3a (Abcam, USA), β-catenin (CST, USA), TGF-β2 (Abgent, USA), COLI (Abcam, USA) GAPDH (BOSTER, China), respectively. Then, the membranes were washed and incubated with horseradish peroxidase-conjugated Goat anti-Rabbit IgG antibody (Abgent, USA). The blots were visualized with chemiluminescence.

### SFRP2 intervened the expression of wnt/β-catenin signaling pathway in ADSC treated photoaging HDF

On the basis of the document and previous experiment,injected 50 ng/ml,100 ng/ml,150 ng/ml final concentraion of the SFRP2 into ADSC-CM co-cultured photoaging HDF, 37°C 5% CO_2_ constant temperature cultured,after 48 h, measureed wnt3a and β-catenin expression in mRNA level.

### Statistical analysis

Data were collected from at least three independent experiments. One-way ANOVA test, followed by paired t-test, was used for statistical analysis among different groups. P < 0.05 was considered significant, p < 0.01 was considered significant obviously.

## Competing interests

The authors declare that they have no competing interest.

## Authors’ contributions

XX carried out most of the experiments and drafted the manuscript; WHY carried out the in vivo studies; ZY and LYQ assisted in the in vitro and vivo studies; LY participated in the design of the studies; JJD and TK participated in the design of the studies; WCT participated in the design of the studies; LXY conceived the studies, coordinated the experiments and was involved in the drafting the manuscript and revising it. All authors read and approved the final manuscript.
